# Sensory properties of selected biofortified common bean (*Phaseolus vulgaris*) varieties grown in Burundi

**DOI:** 10.1002/fsn3.3988

**Published:** 2024-02-13

**Authors:** Mary W. Muroki, Lydiah M. Waswa, Robert Fungo, Andrew Kabwama, Nduwarugira Eric, Ntukamazina Nepomuscene, Blaise Ndabashinze, Symon M. Mahungu

**Affiliations:** ^1^ Department of Dairy, Food Science and Technology Egerton University Nakuru Kenya; ^2^ Department of Human Nutrition Egerton University Nakuru Kenya; ^3^ Alliance of Bioversity International and International Centre for Tropical Agriculture (CIAT) Nairobi Kenya; ^4^ School of Food Technology, Nutrition, and Bio‐Engineering Makerere University Kampala Uganda; ^5^ Alliance of Bioversity International and International Centre for Tropical Agriculture (CIAT) Kampala Uganda; ^6^ Institut des Science Agronomiques du Burundi (ISABU) Bujumbura Burundi

**Keywords:** biofortified, consumer preferences, cooked dry common bean, sensory properties, varieties

## Abstract

The dry common bean is an important grain legume used for human consumption worldwide. In Eastern Africa, Burundi has a significantly high per capita consumption of the crop. There has been significant research on the underlying agronomic traits of dry biofortified common beans, such as disease resistance. However, there is limited systematic information describing the sensory properties of these bean varieties, particularly in Burundi. This study evaluated the sensory properties of eight cooked dry biofortified common bean varieties using a panel of fifty‐four (fourteen plus forty) persons for descriptive sensory evaluation and consumer acceptability tests. Kinure, a traditional non‐biofortified common bean variety, was the control. Based on differences in the attributes of the bean varieties, two‐dimensional principal component analysis (PCA) explained 58.94% of the variation. The attributes of astringency, consistency, color, juiciness, beany aroma, stickiness, and bean size contributed mostly to the differentiation of the bean varieties. A 95% PCA prediction ellipse displayed stronger congruity in the descriptive attributes of NUV130, NUV91, RWV1129, RWV1272, and RWR2245. In contrast, a deviation in the descriptive attributes of MAC44, MAC70, and RWR2154 was discerned. Regarding consumer acceptability tests, the varieties RWR2245 and MAC44 garnered significantly higher (*p* < .05) sensory scores on color, aroma, taste, texture, and overall acceptability. Therefore, the physical traits of cooked biofortified common bean varieties are a major contributor to varietal disparities in consumer acceptance studies. These parameters can greatly impact the adoption of dry biofortified common beans and could be of concern to common bean breeders.

## Graphical abstract

Based on differences in the attributes of the biofortified common bean varieties, two‐dimensional principal component analysis (PCA) explained 58.94% of the variation. The attributes of astringency, consistency, color, juiciness, beany aroma, stickiness, and bean size contributed mostly to the differentiation of the bean varieties. A 95% PCA prediction ellipse displayed stronger congruity in the descriptive attributes of NUV130, NUV91, RWV1129, RWV1272, and RWR2245. In contrast, a deviation in the descriptive attributes of MAC44, MAC70, and RWR2154 was discerned.

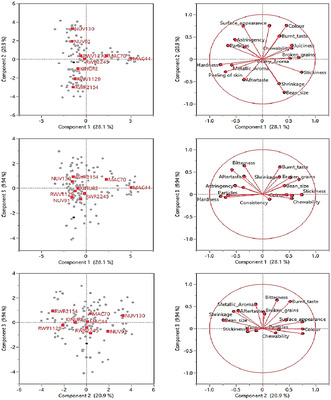

## 1 INTRODUCTION

Common beans (*Phaseolus vulgaris*) are grain legumes belonging to the Leguminosae family and are estimated to be the most important legume utilized for human consumption globally (Amongi et al., 2018). Common beans also belong to a group of foods referred to as pulses that include other edible leguminous crops such as cowpeas, black beans, and chickpeas (Hayat et al., 2014). These foods are mostly consumed in the form of seeds (mature or immature, green or dried), but they can also be utilized as vegetables (both leaves and pods) (Buruchara et al., 2011; Katungi et al., 2020). The fresh form of the bean grain is most preferred because of its fresh flavor and good taste, and it requires a considerable amount of cooking time (approximately 40 min) (Loko et al., 2018). However, storing fresh beans is challenging, making their consumption only possible while in season before the beans dry (Loko et al., 2018).

Biofortified crops are typically consumed by all household members, thus providing nutrients to a larger population, including those who cannot economically access commercially fortified foods (Gilligan, 2012). Biofortified common beans are not only rich in macronutrients such as proteins, carbohydrates, and substantial amounts of lipids (Hayat et al., 2014), but also micronutrients such as minerals, particularly iron and zinc (Mughi, 2017). In Burundi, common beans provide 20% and 50% of the required calories and protein needs (Katungi et al., 2020). Furthermore, in Eastern and Southern Africa regions, particularly Kenya, Tanzania, and Malawi, common beans are usually prepared in a wide range of recipes, thus contributing to food and nutrition security (Katungi et al., 2009).

A major challenge in crop biofortification programs is the acceptance of biofortified crops, especially when changes in appearance characteristics such as the color of the staple food are visible (Hotz & Mcclafferty, 2007). Moreover, the success of any crop biofortification program lies primarily in the consumer's acceptance of the particular crop (Oparinde et al., 2015). Acceptance and adoption of biofortified common bean varieties means that the beans can be grown for a longer time and are hence more sustainable in the long run in the alleviation of malnutrition (De Steur et al., 2012; Gilligan, 2012). Furthermore, having common beans with improved agronomic aspects while maintaining or enhancing their organoleptic properties may lead to profitable seeds for farmers and the homogenecity of products made from the common beans (Almirall et al., 2010; Sparvoli et al., 2021).

Thermal processing is the most crucial step undertaken before the consumption of common beans. Cooking is performed to achieve palatability and to inactivate toxic compounds such as lectins and phytohemagglutinin, a protein that is considered poisonous when consumed either in raw or undercooked beans (Kumar et al., 2013; Li et al., 2022). Additionally, the cooking process imparts sensory properties of common beans, including texture, flavor, and appearance, which are evaluated for quality determination (Shin et al., 2013). Sensory analyses act as a direct means to evaluate the product's qualities, including texture, flavor, color, and overall acceptability. The flavor attribute is usually a combination of taste and aroma (Roland et al., 2017). Pulses are characterized by off‐flavors that are partially inherent and partially produced during harvesting, processing, and storage. These off‐flavor compounds contribute generously to the flavor (taste and aroma) perception and overall acceptability of pulses, and a comprehensive review of this aspect has been discussed by Roland et al. (2017).

Texture is assessed through subjective and objective hardness measurements, while flavor perception uses a more complex multimodal nature that includes olfaction, taste, somatosenses, and other sensory elements processed by the brain (Bi et al., 2021). While descriptive sensory evaluation is used to determine the nature of the differences between products (Oh et al., 2022), determining consumer preferences or acceptability of the products is potentially useful in directing the production and marketing industries toward a particular food or food product for commercial success (Sparvoli et al., 2021). Varieties with thin, soft seed coats are associated with less cooking time and have soft gravy aspects that are attractive to most consumers (Katungi et al., 2009). Furthermore, most bean consumers prefer beans that are light‐colored to dark‐colored beans (Erfatpour & Pauls, 2020). Dark‐pigmented beans are related to old beans that are harder to cook and require a lot of energy to prepare a meal, while light‐colored beans are also associated with freshness and quality seeds (Erfatpour & Pauls, 2020).

It is often a challenge to incorporate sensory properties into common bean breeding programs since most programs usually focus on agronomic traits such as disease resistance, maturity rates, yields, and various nutritional components. Substantial scientific research has been done on the determination of cooking properties, physicochemical properties, and sensory properties of biofortified common bean varieties and bean products in various African countries, particularly in regions under the Pan African Bean Research Alliance program (Byarugaba et al., 2020; Gama et al., 2022; Mughi, 2017; Oparinde et al., 2015). However, there remains limited information on the sensory properties of selected biofortified dry common beans grown and consumed in Burundi.

In light of this information, the current study aimed to evaluate the sensory attributes and consumer acceptability of selected dry biofortified common bean varieties that have been widely released, cultivated, sold, and consumed in Burundi. These varieties were developed through conventional breeding practices. Results obtained from this study can pave the way for the identification of important sensory descriptors that determine consumer preferences and the acceptability of dry biofortified common bean varieties. Furthermore, information on these parameters will inform bean breeders and other stakeholders in the common bean value chain of major gaps regarding dry biofortified common bean varieties.

## 2 MATERIALS AND METHODS

### 2.1 Experimental sites

Nine (*n* = 9) different varieties of common bean were purposively obtained from farmers working with the Pan African Bean Research Alliance (PABRA) through the Institut des Sciences Agronomiques du Burundi in Bujumbura, Burundi. These varieties are new and have been bred and released to farmers in Burundi under the Pan African Bean Research Alliance program in the country. These bean varieties were selected because they are commonly grown, widely consumed in Burundi, have a high concentration of iron and zinc, and were also available at the time of the study. The bean samples were packed in Purdue Improved Crop Storage (PICS) bags and transported to the food chemistry laboratory at Egerton University. Bean samples were then prepared in the food chemistry laboratory plant, and sensory analyses were conducted in the sensory evaluation room of the Guildford Institute at Egerton University's Department of Dairy, Food Science, and Technology. The sensory room consists of individual testing booths illuminated by white light.

### 2.2 Preparation procedures for common beans

Grains of the different dry biofortified common bean varieties were cooked according to Romero del Castillo et al. (2012), with modifications whereby granite pots were used instead of stainless‐steel pots. The dry biofortified common bean varieties included: RWV1129 (Murengeti), RWV1272 (Mutwenzi), MAC44 (Magorori), and MAC70 (Rwizibigega) – released in 2015; RWR2245 (Kaneza), and RWR2154 (Murwiza) – released in 2016; and NUV130 and NUV91 – released in 2018 (Buruchara, 2019). Each variety's bean grains (250 g) were soaked overnight in distilled water in a 1:4 (w/v) ratio (bean weight to distilled water). The soaking water was then discarded, and regular pot cooking was undertaken in granite cooking pots with half‐open glass lids at temperatures of 93.5°C (the boiling temperature of water in Njoro). The cooking time ranged from 60 to 110 min. Hot distilled water was added to correct the water lost by evaporation during the boiling process. Considering the dry biofortified common beans utilized in this study are high in iron and zinc, distilled water was used to prevent contamination with minerals from tap water, which would interfere with the descriptive sensory traits of the cooked biofortified common beans varieties. Cooking was determined by each variety's morphology, color, and soft texture, and through hand pressing to check for a soft texture characteristic of cooked common beans.

### 2.3 Ethical approval

This study was reviewed and approved by the Egerton University Research and Ethics Committee and the National Commission for Science, Technology, and Innovation, Kenya, under License No. NACOSTI/P/21/14502. Written informed consent of the sensory panelists, who comprised students and staff from the Department of Dairy, Food Science, and Technology at Egerton, was obtained before participation in the study. These participants were briefed on the study objectives and protocols and asked if they were willing to participate after addressing all their concerns. Furthermore, informed consent was obtained from all the participants: “I am aware that my responses are confidential, and I agree to participate in this survey and that I can withdraw from the survey at any time without giving a reason. The appropriate protocols for protecting the rights and privacy of all participants during the execution of the research were utilized. All the experiments were performed per relevant guidelines and regulations.

### 2.4 Sensory panel selection

A total of fifty‐four (*n* = 54) panelists were selected to participate in the study. Fourteen (*n* = 14, trained panelists) students and staff in the Department of Dairy, Food Science, and Nutrition at Egerton University were recruited to participate in the descriptive analyses study, and forty other (*n* = 40, semitrained) persons were randomly selected for participation in the consumer acceptability study. Participation as a panelist in the descriptive sensory analyses of cooked dry biofortified common bean grains was based on the frequency of bean consumption (twice per week), willingness to consume beans, sensory acuity determined by the basic taste recognition test and triangle test (Meilgaard et al., 2007), some experience in descriptive sensory evaluation, no food allergies, and availability. Selection and screening of the sensory panel were then carried out as described by Syeunda et al. (2021). The panelists were then invited to participate in the study via phone calls, text messages, and emails. All panelists were also screened by identifying sensory attributes that describe the taste, flavor, and appearance of different cooked bean varieties (Meilgaard et al., 2007).

#### 2.4.1 Descriptive sensory analyses panel training

The final panel of 14 judges consisted of 8 men and four women in the age range of 24–35 years. The panelists were trained by a sensory expert as described by Byarugaba et al. (2020) and as per the generic descriptive approach described by Meilgaard et al. (2007). Panelists were also exposed to the cooked common bean to be tested in the analysis sessions. The panelists generated terms (descriptors) and definitions through consensus to describe the sensory differences perceived among the cooked dry common bean samples. A thorough literature review, coupled with panelist experiences and suggestions, was finally used to develop and refine basic descriptors following the example of a consensus technique described by Oh et al. (2022). Panelists also decided on words to anchor the descriptive terms and some reference standards to be used for the cooked bean samples. Finally, a final sample reference, inclusive of the various terms generated by the panelists, was compiled, along with references with known examples to suit the descriptors reviewed. Table 1 shows the final determined descriptor list. The panel of judges was then shown how to rate the intensity of each attribute using a nine‐point hedonic scale to achieve an ideal alignment throughout the panel. These activities were carried out in multiple group practice sessions before the actual evaluation day. Trial evaluations were conducted, and panelist performance was tested to ensure reproducibility as described by Byarugaba et al. (2020).

**TABLE 1:** Descriptors, their definitions, references, and rating scale used to explain the perception of the selected cooked dry biofortified common bean varieties.

Descriptor	Definition	Reference	Rating scale
Appearance			
Color	The intensity of color ranges from light amber/brown to dark amber/brown.	1 = light brown (mafuko packaging bag) 9 = dark brown (dark mahogany color)	1 = light brown 9 = dark brown
Consistency	The extent of visual uniformity in the bean skin	1 = cheetah's skin 9 = uniformly distributed	1 = patchy 9 = uniformly distributed
Surface appearance	The extent of the shininess of the surface of the bean as a result of boiling the beans	1 = the skin of green bananas 9 = green banana leaf	1 = dull 9 = glossy
Broken grains	Bean grains are broken by the cooking process.	1 = young green beans 9 = dry cooked bean grains	1 = less 9 = most
Shrinkage	Degree of shrinkage on the bean grains' surface	1 = smooth bean grain (unsoaked) 9 = shriveled bean grain(soaked)	1 = least 9 = most
Bean size	Size of the bean grains	1 = wairimu beans 9‐grains bigger than Nyayo beans	1 = small 9 = big
Aroma			
Overall aroma intensity	The intensity of the beany aroma associated with cooked beans	1 = strange aroma 9 = natural aroma of cooked beans	1 = less intense 9 = most intense
Burnt	The aroma of burnt beans	1 = freshly cooked beans 9 = beans with a smoky burnt smell	1 = less intense 9 = most intense
Metallic Aroma	Associated with the environment of bean cultivation	1 = freshly squeezed orange juice 9 = rusted iron	1 = less intense 9 = most intense
Taste and flavor			
Beany flavor	The intensity of flavor characteristic of cooked beans	1 = off odor 9 = natural flavor of cooked beans	1 = less perceivable 9 = highly perceivable
Burnt taste	Characteristics of burnt‐cooked beans	1 = well‐cooked bread crust 2 = burnt bread crust	1 = less intense 9 = more intense
Metallic flavor	Associated with cooking beans in metallic irons or growth habitat	1 = natural flavor of cooked beans 9 = rusted iron	1 = less intense 9 = more intense
Starchy	The extent of taste associated with high‐starch foods, especially when raw starch is consumed.	1 = vegetable salad 9 = boiled cassava	1 = less intense 9 = more intense
Sweetness	The intensity of the taste of sweetness associated with sweet beans	1 = less sweet 9 = very sweet	1 = less intense 9 = more intense
Astringency	The extent of puckering/shrinking, or drying sensation on the surface and/or edges of the lips, tongue, and mouth	1 = unfermented porridge 9 = lemon juice	1 = less intense 9 = more intense
Lingering/aftertaste	The intensity of the sensation after swallowing, i.e., how long the sensation lasts in the mouth.	1 = taste of cooked beans 9 = aftertaste of raw beans	1 = less intense 9 = more intense
Texture			
Juiciness	Quantity of water liberated during chewing	1 = boiled cassava 9 = melon	1 = least juicy 9 = very juicy
Chewability	How chewable are the bean grains	1 = Puffed snacks (wow wow) 9 = cooked meat	1 = least chewable 9 = highly chewable
Hardness	The force with which samples rapture at the first bite	1 = freshly baked bread crumb 9 = roasted groundnuts	1 = least hard 9 = very hard
Grittiness/sandiness	The extent of small, hard particles between teeth during chewing	1 = freshly cooked beans 9 = a mixture of well‐cooked beans and half‐cooked beans	1 = none 9 = many
Particles	The extent to which particles are felt in the mouth after swallowing	1 = sliced mangoes 9 = well‐stored cookies	1 = least 9 = many
Stickiness	The degree to which residues adhere to the teeth during and/or after chewing.	1 = water 9 = tofi/eclairs sweets	1 = less sticky 9 = highly sticky
Sliminess	A slimy mouth feel	1 = cooked mashed potatoes 9 = murenda vegetables	1‐least slimy 9 = very slimy
Peeling of the bean skin	Peeling of the skin while chewing cooked bean grains	1 = boiled green maize 2 = boiled dry maize	1 = least 9 = high

### 2.5 Descriptive analysis procedure

The fourteen panelists rated the attribute intensities of the nine cooked dry common bean varieties on a questionnaire based on a nine‐point hedonic scale by Balthazar et al. (2018), where 1 was “dislike extremely” and 9 was “like extremely.” Each panelist was presented with three boiled common bean samples in three consecutive phases, with a five‐minute break between the phases. Each panelist was also provided with a glass of water for rinsing the mouth before and in between tasting the samples. The cooked common bean samples were coded and randomly served to each panelist at about 50°C. The panelists evaluated the intensity of the sensory attributes based on twenty‐four descriptive terms under the categories of appearance, aroma, taste and flavor, aftertaste, and texture as represented in the lexicon. A two‐minute rest was enforced between sample evaluations to minimize any carryover effect. The bean samples were evaluated in three sessions on three consecutive days, with these sessions acting as replicates (Byarugaba et al., 2020).

### 2.6 Acceptance testing

Nine cooked dry common bean varieties were evaluated for acceptability qualities and rated as per a seven‐scale hedonic test (where 7 = like extremely, 6 = like moderately, 5 = like slightly, 4 = neither like nor dislike, 3 = dislike slightly, 2 = dislike moderately, and 1 = dislike extremely) for a set of attributes vis‐a‐vis, i.e., general acceptability, color, flavor, texture, and taste. The cooked common bean samples were served to the forty participants (18 males and 22 females) aged between 20 and 35 years using a completely randomized design (Lawless & Heymann, 2010).

### 2.7 Principal component analysis (PCA)

Principal component analysis was applied to cluster the descriptive sensory attributes of the cooked dry common bean by studying the correlations among the responses based on an Eigenvalue of above 1.0. The variables were appearance, aroma, taste, and flavor, and texture, with different components under these four main variables as shown in Table 1. Correlation coefficients were applied to form the matrix and extract the Eigenvalue (Demšar et al., 2013).

### 2.8 Experimental design and statistical analysis

The statistical evaluation employed in this study was a completely randomized design (CRD) with 9 factors (8 cooked dry biofortified and 1 non‐biofortified common bean varieties). Data obtained were analyzed using the PROC GLM procedure of the Statistical Analysis System (SAS Institute Inc., 2006) software Version 9.1 and SAS John's Macintosh Project (JMP). An analysis of variance (ANOVA) was performed to test for significance at a 5% confidence level. Tukey's Honestly Significant Difference (HSD) method was employed to perform means separation. The results are expressed as the mean ± standard deviation of three replication measurements. Principal component analysis (PCA) was also employed to measure descriptive sensory attributes, according to Selvaraj et al. (2023). The main intention of using PCA was to ensure data compression and to examine the number of components required for describing the superior part of variation among the cooked dry bean varieties with less information loss (Demšar et al., 2013). The mean attribute ratings listed in Table 6 aid in simplifying the interpretation of data from the 18 attributes (selected using the Kaiser–Meyer–Olkin Measure of Sampling Adequacy criterion) measured on the nine cooked dry common bean varieties.

## 3 RESULTS AND DISCUSSION

### 3.1 Lexicon development and selection of attributes

Lexicon development is a crucial component of sensory studies. This activity entails training panelists using clear references for the various levels of intensity of the attributes to be quantified and arriving at a consensus to enable an accurate description of the food product among the panelists (Romero del Castillo et al., 2008). After various sets of discussions, the panelists agreed to different sub‐groups of attributes describing the appearance, aroma, taste, and flavor, and texture (Table 1). The panel of judges agreed that physical traits such as visual appearance (surface appearance and uniformity of bean skin, broken grains), bean grain size, texture, and color are important traits that determine the acceptability and marketability of the final cooked dry beans (Schoeninger et al., 2017). Furthermore, the panelists decided to separate the beany, burnt, and metallic aroma and the beany, burnt, and metallic flavor during the description analyses to get a distinct difference between the two attributes. Panelists agreed that textural attributes would include juiciness, chewability, hardness, grittiness/sandiness, stickiness, and sliminess (see Table 1). Juiciness, chewability and hardness were perceived as important in describing the cookability of the dry‐cooked common beans while gritiness, stickiness and sliminess would aid in describing the perceptibility of the seedcoat which affects the overall quality and acceptability of common beans (Rivera et al., 2013; Schoeninger et al., 2017). These attributes were considered important quality aspects valued by common bean consumers (Romero del Castillo et al., 2008). The references used for describing the various attributes were items that the panelists were conversant with and made accessible to the team during the training sessions, an activity similar to that of Romero del Castillo et al. (2008).

### 3.2 Descriptive sensory analyses

Tables 2, 3, 4 present the results of the descriptive sensory analysis of the nine cooked dry common bean varieties. The attributes were grouped into appearance, aroma, texture, taste, and flavor. These attributes differed significantly among the varieties (*p* < .05). According to Erfatpour and Pauls (2020), most bean consumers prefer beans that are light‐colored to dark‐colored beans. Dark‐pigmented beans are related to old beans that are harder to cook and require a lot of energy to prepare a meal, while light‐colored beans are also associated with freshness and quality seeds (Erfatpour & Pauls, 2020). The flavor attribute is usually a combination of taste and aroma (Roland et al., 2017). Pulses are characterized by off‐flavors that are partially inherent and partially produced during harvesting, processing, and storage. These off‐flavor compounds contribute generously to the flavor (taste and aroma) perception and overall acceptability of pulses, and a comprehensive review of this aspect has been discussed by Roland et al. (2017).

**TABLE 2:** Appearance and aroma attribute intensity scores for the different common biofortified bean varieties.

Variety	Appearance attributes						Aroma attributes		
	Color	Consistency	Surface appearance	Broken grains	Shrinkage	Bean size	Beany	Burnt	Metallic
Kinure	4.62 ± 0.65^cd^	4.77 ± 0.61^cd^	4.46 ± 0.74^b^	4.00 ± 0.34^bc^	6.38 ± 0.21^a^	6.46 ± 0.35^c^	6.23 ± 0.43^bc^	3.08 ± 0.43^b^	4.00 ± 0.52^bc^
NUV91	6.21 ± 0.52^bc^	7.00 ± 0.59^ab^	7.57 ± 0.36^a^	3.50 ± 0.74^bc^	3.36 ± 0.57^c^	3.21 ± 0.50^de^	5.79 ± 0.54^c^	4.14 ± 0.65^b^	4.29 ± 0.67^bc^
RWR1129	3.36 ± 0.32^de^	3.29 ± 0.32^de^	3.79 ± 0.38^bc^	3.21 ± 0.33^bc^	6.86 ± 0.23^a^	8.43 ± 0.14^ab^	6.43 ± 0.14^bc^	2.86 ± 0.23^b^	4.64 ± 0.23^abc^
RWR2245	5.36 ± 0.43^bc^	6.79 ± 0.49^ab^	4.43 ± 0.50^b^	4.64 ± 0.70^b^	4.00 ± 0.65^bc^	6.00 ± 0.39^c^	6.79 ± 0.49^abc^	3.64 ± 0.59^b^	4.93 ± 0.22^bcd^
NUV130	8.57 ± 0.23^a^	8.07 ± 0.32^a^	8.00 ± 0.21^a^	2.43 ± 0.34^c^	3.86 ± 0.23^c^	1.93 ± 0.22^e^	6.64 ± 0.31^bc^	6.71 ± 0.29^a^	5.07 ± 0.27^ab^
RWV1272	5.86 ± 0.36^bc^	5.21 ± 0.41^bc^	3.93 ± 0.41^bc^	3.79 ± 0.56^bc^	5.79 ± 0.61^ab^	4.21 ± 0.30^d^	5.79 ± 0.61^c^	3.14 ± 0.59^b^	3.57 ± 0.55^bcd^
RWR2154	1.93 ± 0.22^e^	2.07 ± 0.25^e^	2.29 ± 0.19^c^	2.50 ± 0.33^c^	7.14 ± 0.46^a^	8.21 ± 0.21^ab^	6.64 ± 0.32^bc^	3.43 ± 0.34^b^	1.93 ± 0.22^a^
MAC44	8.29 ± 0.22^a^	6.93 ± 0.22^ab^	2.43 ± 0.34^c^	7.29 ± 0.32^a^	6.79 ± 0.42^a^	8.50 ± 0.14^a^	8.43 ± 0.14^a^	8.36 ± 0.13^a^	2.00 ± 0.28^d^
MAC70	7.00 ± 0.28^ab^	4.14 ± 0.27^cd^	5.43 ± 0.14^b^	7.43 ± 0.14^a^	7.00 ± 0.23^a^	7.14 ± 0.23^bc^	7.86 ± 0.23^ab^	2.93 ± 0.22^b^	2.86 ± 0.23^cd^
CV	2.46	2.84	3.18	4.01	2.83	1.84	2.16	3.61	3.92
MSD	1.48	1.66	1.47	2.08	1.96	1.28	1.69	1.84	1.81

*Note*: Mean ratings of the bean varieties with different letters differed significantly (*p* < .05).

Abbreviations: CV, Coefficient of variation; MSD, Minimum significant difference.

#### 3.2.1 Appearance and aroma attributes

Table 2 presents the descriptive intensity scores for appearance and aroma for the eight cooked dry biofortified common beans as scored by the panelists. There was no significant difference (*p* > .05) in color for the MAC70, MAC44, and NUV130 bean varieties, which showed the highest mean score for the attribute, which is 7.00, 8.29, and 8.57, respectively (Table 2). NUV91 and NUV130 had a glossier surface after cooking, while RWR2154 and MAC44 had a dull surface after boiling. Moreover, the surfaces of NUV91 and NUV130 were described as evenly distributed, unlike the surfaces of variety RWR2154, which were patchy. According to Mwangwela et al. (2021), most bean consumers in the rural areas of Malawi preferred biofortified beans that were brown or red. This observation explains why bean varieties with a dark brownish to reddish color (MAC70, MAC44, and NUV130) in the present study scored highly in this attribute and could thus be more attractive to consumers. Physical traits such as visual appearance and grain size, damage level, texture, and color are also important traits that influence the acceptability and marketability of the final cooked beans (Schoeninger et al., 2017).

Broken grains characteristic of green cooked beans were least common in the RWR2154 and NUV91 varieties but highest in the MAC44 and MAC70 varieties. According to Aghkhani et al. (2012) and Gathu et al. (2012), most bean consumers prefer moderately cracked beans and beans with a soft texture. Bean varieties with most split beans and with a soft texture were most preferred by bean consumers in the rural areas of Malawi (Mwangwela et al., 2021), and therefore MAC44 and MAC70 varieties would be most preferred among bean consumers due to this textural aspect.

Bean shrinkage as a result of soaking and cooking was insignificant for the control variety (Kinure) and also for RWV1129, RWV1272, RWR2154, MAC44, and MAC70 biofortified common bean varieties. However, scores for bean shrinkage were highest in MAC70 and RWR2154 at 7.00 and 7.14, respectively, and lowest in the NUV91 bean variety at 3.36. The MAC44 bean variety scored highest in terms of bean size, although the bean size was not significantly different (*p* > .05) for the MAC70 and RWV1129 bean varieties. The NUV130 (1.93) bean variety had the smallest bean size, followed by the NUV91 (3.21) bean variety. MAC44, MAC70, and RWR1129 have a larger bean size compared to NUV91 and NUV130 which are relatively small‐seeded varieties.

Table 2 further shows that the beany aroma associated with cooked beans and the burnt aroma associated with burnt beans were most intense in variety MAC44 (8.43 and 8.36, respectively). There was no significant difference (*p* > .05) in the beany aroma for varieties MAC70 (7.86), MAC44 (8.43), and RWR2245 (6.79). The burnt aroma, however, was significantly different for the MAC44 (2.00) and NUV130 (5.07) varieties. The cooked legumes' beany aroma (green and earthy) characteristic may be attributed to enzymes, non‐enzymes, and chemical reactions induced during heat processing (Bi et al., 2021; Mishra et al., 2019). According to Chigwedere et al. (2019), cooked bean aroma results from a chemical reaction known as strecker degradation. This reaction involves amino acids reacting with a carbonyl 1 compound to form ketones, aldehydes, and other volatile compounds. The more amino acids and carbonyl groups in a bean variety, the more volatile compounds are produced, resulting in higher aroma intensity (Chigwedere et al., 2019).

In the present study, the natural aroma of cooked beans was perceivable in all the cooked dry biofortified common bean varieties. Sensory perception associated with the beany flavor plays a crucial role in consumer acceptance of beans (Bi et al., 2021). Besides the flat or “off” flavor possessed by raw legumes, there is usually an increase in flavor complexity after processing. Heat processing is one of the major processing methods that are used to eliminate the unacceptable flavors in raw beans and contribute to new acceptable flavors (Bi et al., 2021; Chigwedere et al., 2019).

Table 2 also shows that the metallic aroma associated with the beans' cultivation environment was most intense in the NUV130 (5.07) bean variety and least intense in the variety RWR2154 (1.97). It has been reported that dimethyl trisulfide is responsible for the metallic aroma descriptor characteristic of common beans (Cai et al., 2021). Additionally, other research works have shown that other compounds such as 3‐furanmethanol, 2‐ethyl‐6‐methylpyrazine, 1‐octen‐3‐ol, trimethylpyrazine, thymol, 2‐ethyl‐3‐methylpyrazine, and 3‐isopropyl‐2‐methoxypyrazine provide earthy or metallic aroma characteristics in legumes (Bi et al., 2021; Mishra et al., 2019).

#### 3.2.2 Taste attributes

Table 3 shows the taste intensity scores for the different cooked dry common bean varieties. There was no significant difference in the beany taste of the MAC70, MAC44, and NUV130 bean varieties (Table 3). NUV130 had the highest mean score (7.14) for burnt taste, while the RWV1129 variety scored the least (2.57). NUV130 had the highest score for metallic taste (7.14) while RWR2154 had the least score (4.36) for this attribute. From the present study, we can conclude that the aroma of these beans consequently impacted their beany and metallic tastes. Bean varieties that scored highly in beamy aroma (MAC70 and MAC44) had a higher score for beany taste, whereas varieties that had high and low scores for metallic aroma (NUV130 and RWR2154, respectively) had a similar trend in this attribute.

**TABLE 3:** Taste attributes intensity scores for different bean varieties.

Variety	Beany	Burnt	Metallic	Starchy	Sweetness	Sourness	Bitterness	Astringency	Aftertaste
Kinure	5.62 ± 0.65^cd^	3.69 ± 0.51^b^	3.46 ± 0.35^cd^	5.69 ± 0.36^b^	6.08 ± 0.24^a^	3.23 ± 0.30^abc^	2.31 ± 0.35^abc^	3.15 ± 0.37^b^	6.54 ± 0.37^bcd^
NUV91	6.07 ± 0.62^bcd^	3.93 ± 0.55^b^	3.93 ± 0.44^bc^	5.93 ± 0.22^b^	5.14 ± 0.25^a^	4.21 ± 0.47^a^	2.00 ± 0.21^bc^	4.57 ± 0.34^a^	5.93 ± 0.20^cd^
RWV1129	7.07 ± 0.25^abc^	2.57 ± 0.29^b^	3.43 ± 0.39^cd^	6.43 ± 0.23^ab^	3.50 ± 0.42^b^	2.36 ± 0.36^bcd^	1.71 ± 0.22^bc^	2.21 ± 0.35^bc^	7.64 ± 0.23^ab^
RWR2245	6.29 ± 0.58^bcd^	3.21 ± 0.55^b^	4.93 ± 0.22^ab^	6.79 ± 0.21^ab^	6.07 ± 0.22^a^	2.00 ± 0.28^cd^	2.36 ± 0.31^abc^	2.71 ± 0.32^b^	7.00 ± 0.23^abc^
NUV130	7.64 ± 0.31^ab^	7.14 ± 0.23^a^	5.93 ± 0.22^a^	6.50 ± 0.29^ab^	3.57 ± 0.31^b^	2.00 ± 0.21^cd^	3.50 ± 0.33^a^	3.36 ± 0.36^ab^	6.14 ± 0.23^cd^
RWV1272	5.50 ± 0.59^cd^	3.21 ± 0.60^b^	2.71 ± 0.32^cde^	6.79 ± 0.26^ab^	5.50 ± 0.17^a^	3.14 ± 0.35^abc^	2.86 ± 0.39^ab^	3.07 ± 0.35^b^	4.71 ± 0.32^e^
RWR2154	4.36 ± 0.25^d^	3.43 ± 0.34^b^	1.93 ± 0.22^e^	6.14 ± 0.23^b^	3.21 ± 0.33^b^	3.43 ± 0.36^ab^	2.79 ± 0.47^abc^	2.07 ± 0.22^bc^	7.93 ± 0.20^a^
MAC44	8.50 ± 0.14^a^	6.43 ± 0.14^a^	2.14 ± 0.27^de^	7.50 ± 0.14^a^	5.50 ± 0.14^a^	1.50 ± 0.14^e^	1.43 ± 0.14^c^	1.00 ± 0.00^c^	5.43 ± 0.14^de^
MAC70	7.93 ± 0.25^ab^	5.79 ± 0.24^a^	2.07 ± 0.20^e^	6.14 ± 0.51^b^	3.00 ± 0.23^b^	1.43 ± 0.14^e^	2.21 ± 0.33^abc^	2.21 ± 0.30^bc^	5.93 ± 0.25^cd^
CV	2.51	3.52	3.33	1.68	2.19	4.49	5.06	4.29	1.44
MSD	1.80	1.76	1.30	1.23	1.19	1.35	1.38	1.33	0.95

*Note*: Mean ratings of the bean varieties with different letters differed significantly (*p* < .05).

Abbreviations: CV, Coefficient of variation; MSD, Minimum significant difference.

The MAC44 variety had the highest intensity of starchiness (7.50), unlike the other bean varieties. Sweetness was highly perceivable in the control variety (Kinure 6.08) and variety RWR2245 (6.07), and these intensity scores were not significantly different (*p* > .05), whereas MAC70 scored the least (3.00) in terms of sweetness. Similarly, results from a study by Mwangwela et al. (2021) showed a perceived high level of sweetness in NUA 59, a biofortified bean variety grown in the rural settings of Malawi. Unlike cereals, sucrose is the major sugar in legumes. Thermal heat treatment impacts starch gelatinization, and complex sugars break down into simple sugars such as glucose and fructose, bringing about desirable flavors such as the sweet taste of cooked beans (Mkanda, 2007).

Bitterness was most perceived in the NUV30 (3.50) variety and least perceived in the MAC44 (1.43). Results from a study in Malawi showed a perceived bitter taste (Blair et al., 2010) in NUA 35, a biofortified common bean variety grown in the rural settings of Malawi (Mwangwela et al., 2021). Phenolic compounds and mineral content affect the taste (Roland et al., 2017). The higher mineral content/phenolics in biofortified common beans (e.g., iron) contributes to the bitter taste noticed by consumers in the present study and also in the study conducted in Malawi (Ganesan & Xu, 2017; Tuccillo et al., 2022). According to Ganesan and Xu (2017), dark‐colored seeds tend to have a higher content of phenolic compounds. Phenolic compounds, such as condensed tannins in the seed coats, may contribute to a bitter taste after cooking. Hepho, which are black beans consumed in Ethiopia, are considered bitter when eaten without dehulling, and the bitterness is attributed to the high tannin content in their seedcoat (Tura & Mosisa, 2017). NUV130 is characterized by a darker pigmentation unlike the other biofortified common bean varieties, hence a probable explanation for the variety being ranked as the most bitter, even though bitterness was perceived in all varieties.

In terms of sourness, NUV91 had the highest score, while MAC70 had the least score at 4.21 and 1.43, respectively. In terms of astringency, a kind of puckering or dry sensation on the surface of the lips, tongues, and mouth, the NUV91 (4.57) and NUV30 (3.36) varieties had the highest astringency scores, while MAC44 (1.0) displayed the lowest scores. According to Jaeger et al. (2015), astringency is widely perceived in natural foods, including common beans, and is related to free phenolic compounds, which have previously been termed flavor‐active compounds. The puckering sensation is a result of interactions between salivary proteins and phenolic compounds. However, the threshold of astringency varies based on the type and structure of the phenolic compounds (Huang & Xu, 2021). There was no significant difference in the aftertaste scores for the RWR2154, RWR2245, and RWV1129 bean varieties.

#### 3.2.3 Texture attributes

Table 4 shows the textural attribute intensity scores for the selected cooked dry common bean varieties. In terms of juiciness, the MAC44 bean variety was significantly different (P < 0.05) from all other bean varieties (Table 4). The chewability for varieties RWV1129, NUV130, MAC44, and MAC70 was not significantly different (*p* > .05). The RWV1129 variety recorded the highest score (6.93) for hardness compared to MAC44, which recorded the lowest score (1.64). In terms of grittiness, the extent to which small particles are left between teeth during chewing, the RWR2154 (7.43), NUV91 (6.21), and NUV91 (6.79) bean varieties had more particles compared to other varieties, while the MAC44 (1.50) and MAC70 (1.79) varieties had the least number of particles. RWV1272 (6.57), RWV1129 (7.07), NUV91 (6.43), and NUV130 (7.07) varieties contained high amounts of particles felt in the mouth after swallowing, while MAC44 (1.50) had the least number of particles.

**TABLE 4:** Textural attributes intensity scores for different bean varieties.

Variety	Juiciness	Chewability	Hardness	Grittiness	Particles	Stickiness	Sliminess	Peeling skin
Kinure	4.23 ± 0.23^d^	5.23 ± 0.28^e^	6.54 ± 0.31^ab^	3.46 ± 0.31^b^	4.54 ± 0.33^cd^	6.23 ± 0.30^b^	3.38 ± 0.38^cde^	6.08 ± 0.21^bc^
NUV91	4.71 ± 0.22^cd^	6.71 ± 0.22^bcd^	5.86 ± 0.27^ab^	6.79 ± 0.33^a^	6.43 ± 0.14^ab^	4.57 ± 0.29^d^	2.57 ± 0.31^cde^	5.07 ± 0.22^cd^
RWV1129	5.86 ± 0.21^bc^	7.07 ± 0.25^abc^	6.93 ± 0.22^a^	6.21 ± 0.32^a^	7.07 ± 0.25^a^	5.93 ± 0.22^bc^	2.86 ± 0.33^cde^	8.07 ± 0.22^a^
RWR2245	6.00 ± 0.23^bc^	6.50 ± 0.27^cd^	3.64 ± 0.31^d^	3.00 ± 0.28^bc^	5.57 ± 0.29^bc^	6.00 ± 0.21^bc^	6.57 ± 0.36^a^	6.93 ± 0.25^b^
NUV130	6.64 ± 0.31^b^	7.07 ± 0.25^abc^	6.00 ± 0.23^ab^	6.79 ± 0.21^a^	7.07 ± 0.25^a^	3.43 ± 0.17^e^	3.64 ± 0.36^cd^	5.86 ± 0.21^bc^
RWV1272	5.21 ± 0.37^bcd^	6.79 ± 0.21^bcd^	5.71 ± 0.22^b^	2.07 ± 0.32^cd^	6.57 ± 0.17^ab^	6.07 ± 0.22^bc^	5.79 ± 0.30^ab^	6.07 ± 0.25^bc^
RWR2154	3.79 ± 0.35^d^	5.86 ± 0.27^de^	6.07 ± 0.22^ab^	7.43 ± 0.34^a^	4.00 ± 0.28^d^	5.00 ± 0.28^cd^	2.00 ± 0.31^de^	6.43 ± 0.36^b^
MAC44	8.50 ± 0.14^a^	8.00 ± 0.00^a^	1.64 ± 0.13^e^	1.50 ± 0.14^d^	1.50 ± 0.14^e^	8.50 ± 0.14^a^	1.50 ± 0.13^e^	2.57 ± 0.14^e^
MAC70	6.21 ± 0.75^bc^	7.71 ± 0.32^ab^	1.79 ± 0.24^e^	1.79 ± 0.24^d^	5.93 ± 0.22^b^	6.57 ± 0.36^b^	4.64 ± 0.31^bc^	4.14 ± 0.27^d^
CV	2.32	1.37	1.82	2.42	1.65	1.64	3.25	1.59
MSD	1.61	1.05	1.03	1.23	0.98	1.07	1.35	1.08

*Note*: Mean ratings of the bean varieties with different letters differed significantly (*p* < .05).

Abbreviations: CV, Coefficient of variation; MSD, Minimum significant difference.

Results from a recent study on consumer acceptability of biofortified beans in Malawi showed that the grainy attribute of the beans had the least mean impact in terms of liking (Mwangwela et al., 2021). This finding means that bean varieties with the lowest number of particles felt in the mouth during chewing and after swallowing (MAC44 and MAC70) would be the most preferred by bean consumers. The MAC44 (8.50) variety was the stickiest (the extent to which residues adhere to teeth during or after chewing), while NUV130 was the least sticky. The RWR2245 variety had the slimiest mouth feel (6.57) while the MAC44 variety was the least slimy (1.5). The RWR1129 and MAC44 varieties were significantly different in terms of peeling of the skin while chewing, compared to all other bean varieties. The peeling of the skin is attributable to tougher seed coats that may take longer to disintegrate during chewing (Mwangwela et al., 2021).

The integrity of the seed coat in common beans influences the final texture of cooked beans (Schoeninger et al., 2017). Rigid cell walls in dry beans may affect the swelling, water uptake, and dispersion of starch during cooking, and this can contribute to the hard texture (Chen et al., 2015). Additionally, long storage times, probably in retail or farm stores, in the presence of light, high humidity, and high temperatures, could have also resulted in the hard texture, especially in the MAC70 and MAC40 varieties (Schoeninger et al., 2017). The peeling of the bean skin resulted in residues in the mouth, which could also be attributed to tougher seedcoats.

### 3.3 Principal component analysis

Principal components (PCs) were extracted to describe the selected cooked dry biofortified bean varieties based on their sensory characteristics. Three of the extracted principal components with eigenvalues greater than one and accounting for 58.94% of the total variance in the data set were selected for further analysis (Table 5). The PCs were based on the descriptors' maximum variance values to gain specific knowledge about factors of critical importance among sensorial descriptors (Silvano et al., 2014). The scree plot orders the eigenvalues from largest to smallest. A steep line, followed by a steep curve, is obtained after the third component.

**TABLE 5:** Varimax rotated principal component factor loading for sensory attributes of selected common bean varieties.

Sensory attribute	Principal component		
	Factor 1	Factor 2	Factor 3
Stickiness	0.744862	–	–
Broken grains	0.688162	–	–
Juiciness	0.562290	–	–
Chewability	0.554150	–	–
Beany Aroma	0.526154	–	–
Bean size	–	−0.742470	–
Color	–	0.757854	–
Consistency	–	0.756638	–
Surface appearance	–	0.751776	–
Astringency	−0.520368	–	–
Bitterness	–	–	0.643573
Burnt taste	–	–	0.613196
Metallic Aroma	−0.579192	–	–
Shrinkage	–	−0.546187	–
Aftertaste	–	−0.460529	–
Peeling of skin	−0.697243	–	–
Particles	−0.653021	–	–
Hardness	−0.795017	–	–
The proportion of the total variance	28.1	20.9	9.94
TOTAL	58.94		

Based on eigenvector loadings, the results indicated that the first, second, and third principal components explained 28.1%, 20.9%, and 9.94% of the total variation of the data obtained from cumulative proportion (Table 5). These results are close to those from an earlier study assessing flavor and texture qualities in a dry yellow bean population that indicated an overall variation of 63% for three of its principal components (Bassett et al., 2021). Results from a study by Selvaraj et al. (2023) on the sensory attributes of milk chocolates containing common bean and chickpea powder had a total of eight significant principal components and showed a higher variation of 78.6% in the tested data set. Results from another study by Oh et al. (2022) had two significant principal components and recorded a variation of 89.6% of descriptive sensory data for different types of beans (black, kidney beans, mung beans, soybeans, red beans, and green kernel black beans) and chickpeas cultivated and consumed in Korea. The differences in the different studies can be attributed to the varying number of principal components and assessed sensory qualities.

**FIGURE 1:**
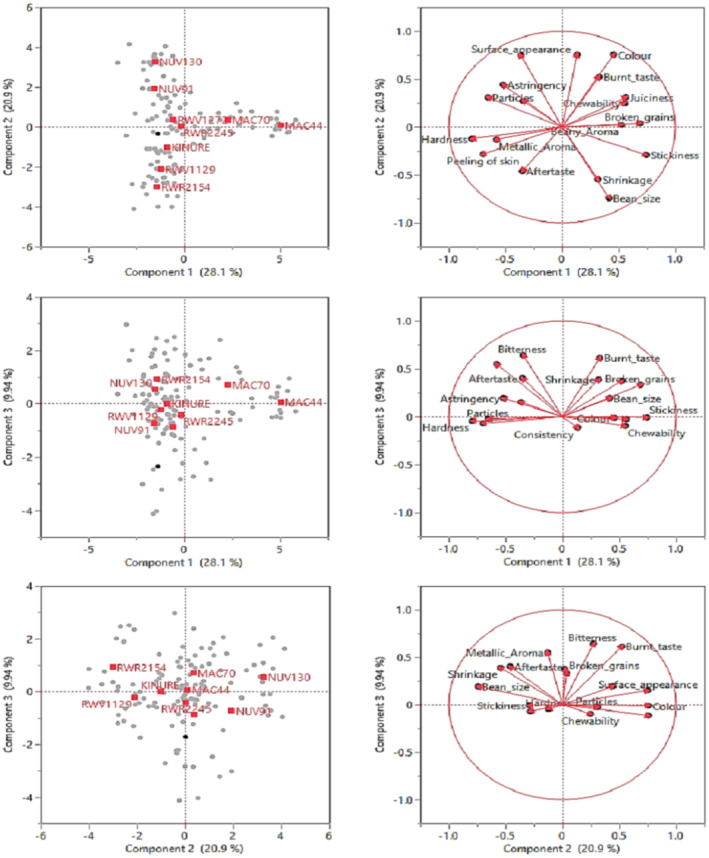
Loading plot of sensory attributes on principal components.

In addition, varimax rotation was applied to these three PCs to get them closer to the original variables, as per Lawless and Heymann (2010). The correlations between PC and the descriptive attribute data are shown by the varimax rotated factor loading (Table 5). The only loadings that were listed were those with an absolute value greater than 0.6, which denoted a strong influence. Moreover, Figure 1 shows the correlation loading plot for the common bean samples for the three PCs and the sensory characteristics. Close measurements are grouped and are positively correlated; far measurements, which are groups of loadings opposite of each other, are negatively correlated and are 180° apart. Measurements that are 90° apart are independent and would therefore be loaded on different PCs (Bassett et al., 2021).

PC1 was strongly correlated to stickiness, broken grains, juiciness, beany aroma, and chewability, thus primarily measuring the enhancement of sensory properties in the various common bean varieties (Table 5). In a different study assessing the sensory attributes of milk chocolate containing different levels of bean and chickpea powder, PC1 was positively correlated to appearance, texture, aroma, and mouthfeel (Selvaraj et al., 2023), an equivalence of broken grains classified under appearance, beany aroma and juiciness, stickiness, and chewability categorized under texture(mouthfeel) in this study. PC1 was also negatively correlated with astringency, metallic aroma, peeling of skin, particles, and hardness. PC2 was positively correlated to color, consistency, and surface appearance and negatively correlated to bean size, shrinkage, and aftertaste (Table 5). Lastly, PC3 was positively correlated with bitterness and burnt taste. In contrast, PC2 was negatively correlated to color and appearance, while PC3 was negatively correlated to taste (Table 5) (Selvaraj et al., 2023).

The loadings of original responses on PC1, PC2, and PC3 are summarized in Figure 1. NUV130 was characterized by surface appearance, hardness, and astringency, while NUV91 was characterized by surface appearance, hardness, and color. NUV130 is dark‐pigmented, an indicator of probable higher free phenolic content than in the NUV91 variety, and this aspect could explain the astringency attribute (Jaeger et al., 2015). Additionally, the lighter pigmentation of the NUV91 bean variety could be a possible explanation for its characteristic color, considering consumers prefer lighter bean seeds to darker ones (Chen et al., 2015). RWV1272 was solely associated with hardness, while RWV1129 was associated with peeling of the skin and aftertaste. Additionally, RWR2245 was characterized by broken grains, whereas RWR2154 was characterized by broken grains, aftertaste, and bean size. Mineral concentration in the seed coat is associated with the splitting of bean grains, and this could be a possible explanation for broken grains, characteristic of the RWV1129 and RWR2245 varieties. Lastly, MAC70 was characterized by a beany aroma, a burnt taste, and astringency, whereas MAC44 was characterized by stickiness, shrinkage, and a burnt taste. The beany aroma in both MAC70 and MAC44 guarantees the bunt taste attribute characteristic of both bean varieties. An earlier study indicated that beany aroma and burnt taste were significantly highly correlated with consumer preferences for bean sauces (Byarugaba et al., 2020). The MAC70 bean variety could thus be utilized in the food industry to develop bean sauces.

Overall, these results indicate that the appearance of common beans, the texture (hardness) of cooked beans, and the taste are the major characteristics sought after by common bean consumers, hence domineering traits in these biofortified common bean varieties. Understanding the effect of each attribute on the liking scores is important not only to bean breeders but also to producers developing bean‐based products (Bassett et al., 2021). Such information provides the possibility to identify key acceptable drivers needed to understand priorities when developing new common biofortified or improved bean varieties and when formulating and reformulating existing common bean products (Jaeger et al., 2015).

### 3.4 Acceptance test

Consumer scores for the sensory acceptability of the cooked dry common bean varieties are presented in Table 6. The overall acceptability score ranged from 4.07 to 5.86 on a 7‐point hedonic scale. These results indicate that the degree of liking of the cooked dry common bean varieties fell under the category “like slightly to neither like nor dislike.” These results are in agreement with the results from studies by O'leary et al. (2009) and Sparvoli et al. (2021), which indicated the overall liking scores for plain boiled soya beans, lentils, and chickpeas, and biscuits made from common bean flour, respectively, to be in the same range. However, both studies scored the sensory attributes on a 9‐point hedonic scale, unlike the current study, which used a 7‐point hedonic scale.

**TABLE 6:** Consumer scores for sensory characteristics for the 9 common bean varieties.

Variety	Color	Aroma	Taste	Texture	General acceptability
RWV2154	4.12 ± 1.80^a^	4.21 ± 1.46^a^	4.10 ± 1.51^a^	4.12 ± 1.27^a^	4.07 ± 1.63^a^
RWV1129	4.71 ± 1.57^ab^	4.67 ± 1.56^ab^	4.74 ± 1.47^ab^	4.76 ± 1.40^ab^	4.95 ± 1.41^ab^
KINURE	4.71 ± 1.42^ab^	4.95 ± 1.38^abc^	5.19 ± 1.40^bc^	4.90 ± 1.51^ab^	5.31 ± 1.37^b^
RWV1272	5.05 ± 1.08^abc^	5.00 ± 1.01^abc^	5.38 ± 1.08^bc^	5.36 ± 1.08^b^	5.36 ± 1.10^b^
MAC70	5.17 ± 1.19^bc^	4.86 ± 1.20^abc^	4.90 ± 1.30^abc^	4.86 ± 1.56^ab^	4.98 ± 1.35^ab^
NUV130	5.26 ± 1.48^bc^	4.74 ± 1.27^ab^	4.62 ± 1.71^ab^	5.19 ± 1.40^b^	5.31 ± 1.41^b^
NUV91	5.29 ± 1.31^bc^	4.74 ± 1.31^ab^	4.93 ± 1.33^abc^	5.02 ± 1.20^ab^	4.95 ± 1.31^ab^
RWR2245	5.55 ± 0.99^bc^	5.21 ± 1.34^bc^	5.48 ± 1.29^bc^	5.19 ± 1.25^b^	5.52 ± 1.21^b^
MAC44	5.67 ± 1.24^c^	5.67 ± 1.36^c^	5.79 ± 1.16^c^	5.50 ± 1.15^b^	5.86 ± 0.84^b^
CV	6.32	7.06	8.85	6.30	5.96
MSD	0.93	0.92	1.38	0.91	0.93

*Note*: Mean ratings of the bean varieties with different letters differed significantly (*p* < .05).

Abbreviations: CV, Coefficient of variation; MSD, Minimum significant difference.

Based on the results presented in Table 6, variety MAC44 (5.86) had the highest overall acceptability score, while variety RWR2154 (4.07) had the least overall acceptability score. Among the other eight cooked dry biofortified common bean varieties, variety RWR2245 had higher scores for most of the sensory attributes apart from texture, which was not statistically different from that of the other varieties, i.e., 5.55 (color), 5.21 (aroma), 5.48 (taste), 5.19 (texture), and 5.52 (overall acceptability). Moreover, compared to the control (Kinure), only two cooked dry biofortified common bean varieties, RWV2154 and RWV1129, scored the least in all their sensory attributes and overall consumer acceptability.

It is worth noting that there was no significant difference in the scores for color and taste in most bean varieties apart from varieties RWR2154 and MAC44, which showed a significant difference in the two attributes. Furthermore, varieties RWV1272, RWR2245, and MAC44, which had higher acceptability scores for color, also garnered higher acceptability scores for taste and overall acceptability. NUV130, RWR2245, RWV1272, and MAC44 had the highest scores for texture and flavor (taste and aroma combined).

Seed coat color plays a crucial role in determining consumer acceptability and the seed quality of dry beans. Additionally, the final bean color is a key parameter appreciated by canned bean producers (Güzel & Sayar, 2012). In the present study, varieties RWR2154 and RWR1129 scored the least in terms of color and are characterized by lighter seed pigmentation, unlike the other bean varieties which are dark‐pigmented. From Table 2 and 6 consumers in the present study preferred common bean varieties that were dark‐colored since they garnered higher scores. Factors such as degradation of pigment, darkening reactions, and heavy metal contamination may alter the final color of processed beans and hence influence consumer acceptability (Güzel & Sayar, 2012).

Furthermore, exposure to light, elevated temperatures, and high humidity can lead to a phenomenon known as seed coat darkening. Darkened seeds are presumed by consumers to be old and hard to cook, hence reducing the commercial viability of such seeds (Chen et al., 2015). It is therefore important to educate farmers and bean traders on the proper storage of common beans to avoid the development of this undesirable trait. Additionally, seed coat post‐harvesting darkening may alter flavor over time. Selection against darkening is possible and should be prioritized when developing new improved and biofortified bean varieties to ensure both greater visual appeal to consumers and a bypass to flavor changes (Bassett et al., 2021; Erfatpour & Pauls, 2020). This impact of color on the taste of dry beans could be the reason why varieties RWV1272, RWR2245, and MAC44 obtained higher acceptability scores for color, taste, and overall consumer acceptability.

Texture attributes such as sliminess, smoothness, and hardness affect the flavor (taste and aroma) of dry beans (Bassett et al., 2021). This finding could be a possible explanation for why varieties NUV130, RWR2245, RWV1272, and MAC44 had the highest scores for texture and flavor. Chemical reactions taking place during thermal processing are responsible for the flavor of cooked beans. It has also been reported that the main compounds that contribute to the flavor of cooked beans are aldehydes, alcohols, ketones, sulfur compounds, and some heterocyclic compounds (Khrisanapant et al., 2019).

## 4 CONCLUSION AND RECOMMENDATIONS

Descriptive analyses indicated that appearance, texture (hardness), and taste of cooked common beans are the major characteristics sought after by common bean consumers. Regarding acceptance analyses, varieties RWR2245 and MAC44 scored greatly in terms of appearance, texture, flavor (taste and aroma), color, and overall acceptability. This observation indicates that it is possible for common bean biofortification programs to produce varieties with both excellent agricultural and nutritional traits while at the same time remaining attractive and acceptable to common bean consumers. The overall acceptability mean score for all the cooked common bean varieties ranged from 4.07 to 5.86 on a 7‐point hedonic scale. The beany aroma and taste and texture of legumes in general impacted their acceptability, a possible explanation as to why overall consumers' acceptability scores of the biofortified common beans were in the range of neither like nor dislike to slightly like.

Boiling was the cooking method utilized in this study and hence provides a basic foundation for sensory evaluation of the biofortified common bean varieties. Therefore, incorporation of other cooking methods, such as stewing with other food ingredients, can help minimize the perception of the beany flavor and taste and hence contribute further to the overall acceptability of these bean varieties. We, therefore, recommend such a study exploring various cooking methods, considering there are various bean‐based products that have already been developed and are already on the market. Additionally, the PABRA program can engage farmers at the household level through nutrition‐sensitive outreaches incorporating different methods of preparing biofortified and improved common beans before consumption.

On the other hand, sensory analyses have certain limitations as they rely on individual sensory preferences. Additionally, there is still much to be understood regarding the flavor and texture of these dry, biofortified common beans. Therefore, other methods for sensory evaluation can be employed to further explain the sensory attributes of these new varieties of biofortified common beans not only in Burundi but in the other PABRA regions. Other methods for assessing these sensory traits, including GC–MS and texture measurements, should be explored. Lastly, considering the sensitive nature of consumer acceptance of biofortified foods, we recommend future bean breeding programs incorporate other methods, such as the use of near‐infrared spectroscopy, for characterizing the sensory quality of beans, especially at the gene bank stage. Understanding which traits are most important for consumer preferences and their expectations for different seed types will help breeders address all these sensory attributes with a focused and more efficient approach.

## AUTHOR CONTRIBUTIONS

**Mary W. Muroki:** Conceptualization (lead); data curation (lead); formal analysis (lead); investigation (lead); methodology (lead); project administration (lead); software (lead); visualization (lead); writing – original draft (lead); writing – review and editing (lead). **Lydiah M. Waswa:** Conceptualization (supporting); methodology (supporting); project administration (supporting); resources (supporting); supervision (equal); validation (equal); visualization (supporting); writing – review and editing (supporting). **Robert Fungo:** Conceptualization (supporting); funding acquisition (lead); methodology (supporting); project administration (equal); resources (supporting); supervision (equal); validation (equal); visualization (supporting); writing – review and editing (supporting). **Andrew Kabwama:** Data curation (supporting); project administration (supporting); resources (supporting); validation (supporting). **Ntukamazina Nepomuscene:** Project administration (supporting); resources (supporting). **Nduwarugira Eric:** Project administration (supporting); resources (supporting); supervision (supporting). **Blaise Ndabashinze:** Investigation (supporting); project administration (supporting); resources (supporting). **Symon M. Mahungu:** Writing – review and editing; supervision; validation; conceptualization.

## FUNDING INFORMATION

This work was made possible by the International Centre for Tropical Agriculture (CIAT) and the Centre of Excellence in Sustainable Agriculture and Agribusiness Management (CESAAM), Egerton University.

## CONFLICT OF INTEREST STATEMENT

The authors declare no conflict of interest.

## Data Availability

The data that support the findings of this study are available on request from the corresponding author.
